# Impaired Representation of Time in Schizophrenia Is Linked to Positive Symptoms and Cognitive Demand

**DOI:** 10.1371/journal.pone.0067615

**Published:** 2013-06-27

**Authors:** Jutta Peterburs, Alexander M. Nitsch, Wolfgang H. R. Miltner, Thomas Straube

**Affiliations:** 1 Institute of Medical Psychology and Systems Neuroscience, University of Muenster, Muenster, Germany; 2 Department of Biological and Clinical Psychology, University of Jena, Jena, Germany; University Of São Paulo, Brazil

## Abstract

Time processing critically relies on the mesencephalic dopamine system and striato-prefrontal projections and has thus been suggested to play a key role in schizophrenia. Previous studies have provided evidence for an acceleration of the internal clock in schizophrenia that may be linked to dopaminergic pathology. The present study aimed to assess the relationship between altered time processing in schizophrenia and symptom manifestation in 22 patients and 22 controls. Subjects were required to estimate the time needed for a visual stimulus to complete a horizontal movement towards a target position on trials of varying cognitive demand. It was hypothesized that patients – compared to controls – would be less accurate at estimating the movement time, and that this effect would be modulated by symptom manifestation and task difficulty. In line with the notion of an accelerated internal clock due to dopaminergic dysregulation, particularly patients with severe positive symptoms were expected to underestimate movement time. However, if altered time perception in schizophrenia was better explained in terms of cognitive deficits, patients with severe negative symptoms should be specifically impaired, while generally, task performance should correlate with measures of processing speed and cognitive flexibility. Patients underestimated movement time on more demanding trials, although there was no link to disease-related cognitive dysfunction. Task performance was modulated by symptom manifestation. Impaired estimation of movement time was significantly correlated with PANSS positive symptom scores, with higher positive symptom scores associated with stronger underestimation of movement time. The present data thus support the notion of a deficit in anticipatory and predictive mechanisms in schizophrenia that is modulated both by symptom manifestation and by cognitive demand.

## Introduction

“The only reason for time is so that everything doesn’t happen at once.” As pointed out in this incisive quote by Albert Einstein, time is a fundamental dimension which helps us organize and make sense of events in our environment. Notably, time perception is highly subjective and may vary as a function of psychological and physiological factors [1]. Interesting tasks are judged as significantly shorter than boring tasks [2], and individuals experiencing low levels of boredom have been shown to underestimate time to a greater extent than highly boredom prone individuals [3]. Recent findings show that time perception is also influenced by action preparation: time is perceived as slowed down during preparation of a ballistic reaching movement, thereby facilitating last-minute adjustments in action execution [4].

How is time perception implemented given that there is no dedicated sense organ? The accumulator-pacemaker model assumes an internal clock in which the number of pulses regularly emitted by a pacemaker is temporarily stored in an accumulator and – at the time of feedback - transferred into reference memory [5], [6]. Proposing distinct clock, memory and decisional stages, this model allows individual differences in time perception to be attributed to alterations in attention, pacemaker speed, memory or decisional stages [7]–[10].

There is compelling evidence for a critical role of the striatal dopaminergic system and striato-prefrontal projections for interval timing (for a review see [11]). Temporal processing can be impaired by targeting dopamine (DA) D2 receptors, with DA antagonists decelerating the speed of the internal clock [12]–[14]. Neuroimaging studies consistently show involvement of basal ganglia (BG), prefrontal cortex (PFC) and posterior parietal cortex (PPC) in interval timing [15], [16]. In line with this, a number of psychiatric or neurological disorders involving fronto-striatal circuits have been shown to be associated with deficits in time perception and temporal processing. Patients with lesions of the frontal cortex exhibit impaired motor-timing performance [17]. Patients with Parkinson’s disease (PD) are impaired both at motor timing and time perception [18], while lesions to the BG appear to only disrupt motor-related timing processes [19].

It has been argued that impairments of time processing also play a key role in schizophrenia [20]. Severe disturbances in the subjective experience of time are a well-known phenomenon in some cases of schizophrenia [21]–[23]. It has even been argued that malfunctions of the patients’ internal clock may be associated with highly confusing symptoms like delusions or hallucinations [24]. Previous studies have reported timing deficits suggestive of an acceleration of the “internal clock” in schizophrenia [25]: patients overestimate interval duration when verbally reporting it [26] or during repetitive tapping [27], and underestimate interval duration during time production tasks [26], [28]. Alternative theories attribute timing disturbances in schizophrenia to cognitive impairment [29] or deficient sensorimotor integration [30]. Generally, patients are more variable in timing intervals than controls [27], [31]–[34], although medication, attention and memory may be confounding factors [29].

It is conceivable that the variability of findings may also relate to differences in symptom manifestation. If the level of striatal DA is directly linked to the speed of the internal clock as suggested by Davis [35], dopaminergic hyperactivity in limbic regions due to increased DA type-2 receptors in the BG which is accompanied by pronounced positive symptoms may result in faster clock speed [25]. Decreased activity of DA type-1 receptors in the dorsolateral PFC which is presumed to be associated with negative symptoms, on the other hand [35], may not affect the internal clock. Alternatively, if time processing impairments in schizophrenia are driven by cognitive deficits, patients with pronounced negative symptoms in whom cognitive dysfunction is reported to be more pronounced [36], [37] may be more severely affected.

The present study aimed to assess the relationship between altered time processing in schizophrenia and positive and negative symptoms in 22 patients with schizophrenia and 22 controls by means of a computerized time estimation paradigm requiring subjects to estimate the time needed for a moving visual stimulus to reach a target position. In order to control for potential executive impairment in patients, processing speed and cognitive flexibility were assessed with the Trail Making Test (TMT) [38] and the Wisconsin Card Sorting Test (WCST) [39]. It was hypothesized that patients – compared to controls – would be less accurate at estimating the movement time. In line with the notion of an accelerated internal clock, patients were expected to underestimate movement time, this pattern being particularly pronounced in patients with severe positive symptoms. If altered time processing in schizophrenia is better explained in terms of cognitive deficits, patients – and particularly those with severe negative symptoms - should be specifically impaired on more cognitively demanding trials, while generally, task performance should correlate with measures of processing speed and cognitive flexibility.

## Methods

### Participants

Twenty-two adult individuals (15 male, 7 female; mean age = 35.68±8.73 years) meeting the Diagnostic and Statistical Manual IV (DSM-IV) [40] criteria for schizophrenia and 22 non-psychiatric control participants (15 male, 7 female; mean age = 32.68±7.61 years) were recruited for participation. The present study was part of a larger investigation on cognitive control processes in patients with depression, schizophrenia and obsessive-compulsive disorder and healthy controls. All patients were outpatients of the Clinic for Psychiatry and Psychotherapy in Jena, Germany, and were assessed using the Structured Clinical Interview for the DSM-IV for Axis I disorders (SCID-I) [41]. Inclusion criteria for patients were fulfillment of the criteria for the diagnosis of schizophrenia according to the DSM-IV, and age between 18 and 60 years. Exclusion criteria for all participants comprised self-reported neurological diseases, left-handedness, and a history of substance abuse, head injury resulting in episodes of unconsciousness or head surgery. For patients only, psychiatric disorders other than schizophrenia led to exclusion from the study, as did a personal or family history of schizophrenia in the control group. Furthermore, all control participants were screened with a German version of the Mini International Interview (M.I.N.I.) [42] in order to exclude the presence of any psychiatric Axis I disorder. All participants had normal or corrected-to-normal vision and hearing acuity.

Current symptom levels in patients were assessed by an experienced clinician using the Positive and Negative Syndrome Scale (PANSS) [43] which contains 30 items requiring current symptoms to be scored between 1 (absent) and 7 (extreme). According to convention, symptom ratings were categorized as reflecting positive symptoms, negative symptoms or general psychopathology. Furthermore, the sum of the three symptom scores was calculated. PANSS ratings were available for all but one patient.

Controls participants were matched to the patients according to gender, age, and IQ. Intellectual performance was assessed using the Mehrfachwahl-Wortschatz-Intelligenztest-B (MWT-B) [44], a German verbal intelligence test requiring subjects to distinguish a real word from 4 non-words in 37 items of increasing difficulty. The number of correctly identified words was recorded. MWT-B data was available for all patients and all but one control participant.

### Cognitive Tests

Processing speed and cognitive flexibility were assessed by means of the TMT [38] and WCST [39]. The TMT requires subjects to connect 25 consecutive target circles as fast as possible without lifting the pen off the paper. In Part A (TMT-A), circles are merely numbered (1–25). In Part B (TMT-B) circles are either numbered (1–13) or labeled with letters (A–J), and subjects are required to connect numbers and letters alternatingly (i.e. 1-A-2-B-3-C, etc.). The time needed to complete each part is recorded. Errors are assumed to be reflected in the time needed to complete the test and thus not recorded separately. While Part A provides a measure of attention, visual scanning and processing speed, Part B possesses greater executive demand. Since overall, performance depends on general psychomotor and processing speed, the difference in completion times between Part A and Part B (TMT-B – TMT-A) is thought to reflect a measure of cognitive flexibility.

The WSCT is an established tool to measure set abstraction, set shifting and cognitive flexibility. The test consists of cards displaying different forms in different colours and numbers. Four stimulus cards are presented, and the subject is instructed to sort the response cards under the matching stimulus cards. After each sort, feedback is provided, allowing the subject to learn the current sorting rule. After ten consecutive correct sorts, the sorting rule is changed without warning. In the present study, the test was continued until either 128 trials or 9 categories had been completed. The number of completed categories and the number of perserverative errors were recorded.

### Experimental Task

**Figure 1:**
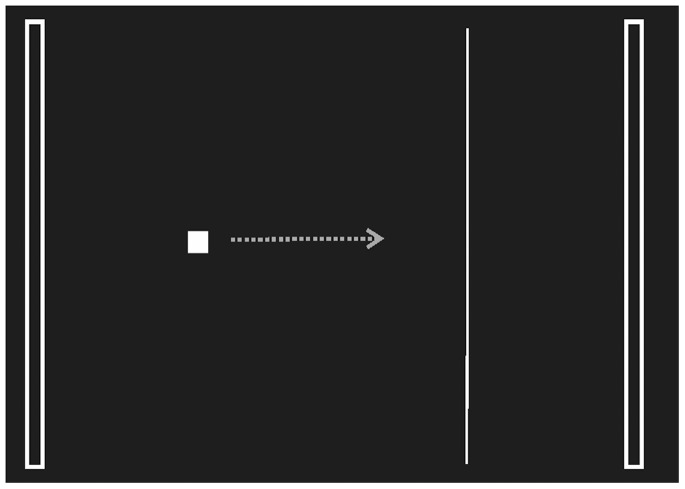
Schematic illustration of the anticipation of movement task. A white square moves horizontally towards a target at slow or fast speed but vanishes before reaching it. A white vertical line marks the vanishing point on 50% of the trials. Subjects are required to indicate anticipated time of arrival by button press.

The anticipation of movement task (AMT) (Nitsch JR, Heinen T, Berger H. Anticipation of Movement Time, Version 1.1. Unpublished computer-assisted test of subjective time representation. Institute of Psychology, German Sport University, Cologne) is a novel task designed to assess visual predictive timing at varying levels of task difficulty. A schematic illustration of the task is provided in Figure 1. A white square moved horizontally from a starting rectangle leftward across a computer screen towards a target rectangle on the right but vanished before reaching it. The vanishing point was marked by a white vertical line on half of the trials. Subjects were required to estimate time of arrival by button press. Two different movement speeds were applied, i.e. completion of the movement takes 3000s or 18000s, and two different positions of the vanishing point, i.e. at 1/3 or 2/3 of the total distance to the target, were balanced across all trials. Absence of the vanishing mark was assumed to increase task difficulty, as disappearance of the white square was not predictable on these trials. Similarly, slow movement speed and longer distances were expected to reflect higher cognitive demand, as mental representations of the movement had to be maintained for longer on these trials. The difference between objective time of arrival (movement time) and estimated time of arrival (reaction time) was recorded on each trial. Larger difference scores reflect less accurate estimations. Positive scores indicate that reaction times were too slow, i.e. movement time was overestimated, negative scores indicate that button presses occurred too early, i.e. movement time was underestimated. No performance feedback on estimation accuracy was provided.

The task comprises a total of 48 trials plus four practice trials at the beginning. A break is introduced after 24 trials. Trial types as determined by presence/absence of the vanishing mark and the two different speeds of movement are balanced throughout the task, and trial order is pseudo-randomized so that each trial type occurs three times before and after the break, respectively. Task completion takes about 20 minutes.

### Procedure

Participants were informed that the study was part of a larger study investigating cognitive control, and specifically the role of the fronto-cingulate system for conflict processing and error monitoring, in patients diagnosed with schizophrenia, depression and obsessive-compulsive disorder. Subjects received monetary reimbursement for participation (8€ per hour).

The study conforms to the Declaration of Helsinki and has received ethical clearance by the Ethics Board of the Medical Faculty at the University Clinic of Jena, Germany. After signing the informed consent form, the experimental task was administered first, followed by the neuropsychological background tests (MWT-B, TMT, WCST). The entire test session took about 45 minutes.

### Statistical Analysis

AMT data were analyzed by means of repeated-measures analysis of variance (ANOVAs) with group (patients vs. controls) as between-subjects factor and speed (slow vs. fast), distance (short vs. long) and line (line vs. no line) as within-subjects factors. Interactions were resolved by means of post-hoc *t* tests. Age, MWT-B, TMT and WCST scores were compared between groups using *t* Tests or Mann-Whitney *U*-Tests, if the requirements for parametric testing were violated. For patients, correlations between cognitive measures and AMT scores and between PANSS and AMT scores were determined by Kendall’s tau (τ). The significance level was set to *p*<.05 one-sided.

## Results

### Clinical and Cognitive Ratings

Table 1 provides an overview of clinical and cognitive ratings for patients and controls. There were no significant group differences with regard to age or IQ (both *p*>.230).

**Table 1:** Overview of mean clinical and cognitive scores for patients and controls.

	Controls	Patients
***PANSS***		
Positive symptoms		17.29 (8.45)
Negative symptoms		18.24 (6.91)
General psychopathology		38.38 (13.77)
Total		73.90 (25.21)
***MWT-B***		
correctly identified words	30.10 (2.57)	29.73 (4.94)
IQ	110.29 (10.24)	112.64 (16.88)
***TMT***		
A (seconds)	28.32 (7.01)	39.36 (11.55)**
B (seconds)	62.27 (28.96)	96.36 (47.77)**
Difference	33.95 (27.41)	57.00 (39.92)**
***WCST***		
Categories	8.27 (1.58)	7.00 (3.18)
Perserverations	22.5 (12.68)	22.73 (14.28)

Standard deviations in parentheses.

** *p*<.01.

Mean reaction times on TMT-A and TMT-B and mean TMT difference scores for patients and controls are provided in Table 1. Patients took significantly longer than controls to complete both TMT-A (*t_(_*_42)_ = −3.833, *p* = .001) and TMT-B (*U* = 101.50, *p* = .001). Accordingly, cognitive flexibility as assessed by the TMT difference score was significantly lower in patients compared to controls (*U* = 114.00, *p* = .003).

There were no significant group differences with regard to the number of categories and the number of perserverative errors on the WCST (both *p*>.189).

### Anticipation of Movement Time

Since the present study is focused on investigating differences in time perception in patients with schizophrenia as compared to non-psychiatric subjects, only the main effects and all interactions involving the factor *group* are reported.

**Figure 2:**
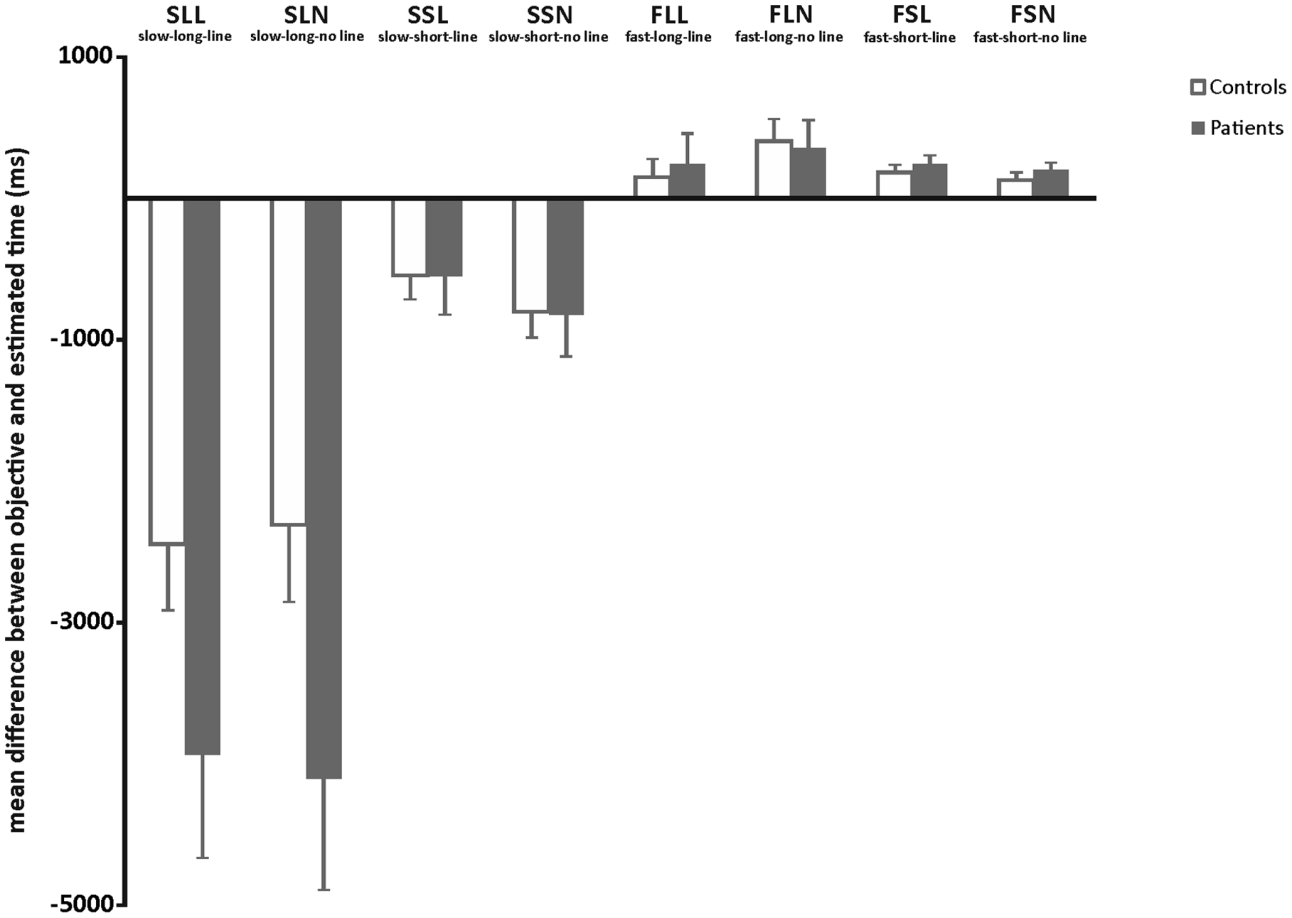
Mean AMT performance in patients and controls. Mean differences between objective and estimated movement time according to target speed (slow/fast), distance (short/long) and line (line/no line) for patients and controls. Positive scores indicate that movement time was overestimated; negative scores indicate that movement time was underestimated. Error bars represent the standard error of the mean.

Mean differences between objective and estimated time according to trial type for patients and controls are provided in Figure 2. The ANOVA yielded significant main effects of *speed* (*F*[_1, 42_] = 73.525, *p*<.0001) and *distance* (*F*[_1, 42_] = 40.296, *p*<.0001), indicating that across all participants movement time was significantly more strongly underestimated for slow movement speeds and for long distances. The main effect of *line* failed to reach statistical significance (*p* = .493), as did the *group x line* interaction (*p* = .299). The *speed x distance x group* (*F*[_1, 42_] = 8.477, *p* = .006) and *distance x group* (*F*[_1, 42_] = 4.810, *p* = .034) interactions were also significant. Post-hoc *t* tests for the three-way interaction comparing AMT scores according to speed and distance conditions (pooled across line conditions) between patients and controls showed that for patients, differences between objective and estimated movement time were significantly more negative only for trials with slow target speed and long distance (*t_(_*_42)_ = 1.841, *p* = .035), indicating that patients significantly underestimated movement time on these trials. After correction for multiple testing (Bonferroni corrected significance level p<.0125), this effect remains a trend only. There were no group differences for the trial types slow-short, fast-long and fast-short (all p>.186). The main effect *group* as well as all other interactions failed to reach statistical significance (all p<.093).

### Correlational Analyses

#### AMT scores and cognitive measures

In order to elucidate a potential relationship between AMT performance and executive function, correlations between AMT and TMT as well as between AMT and WCST measures were calculated separately for patients and controls. Note that since the original analysis had revealed that presence or absence of a line at the target vanishing point did not influence performance, reaction times were pooled across line and no-line trials, rendering the trial types slow-long, slow-short, fast-long, and fast-short. There were no significant correlations between any of the TMT and AMT measures in either group (all *p*>.155). For patients only, a near-significant positive correlation between reaction times on AMT trials with slow speed and short distance and the number of completed categories on the WCST emerged (τ = .273, *p* = .054). None of the other correlations reached statistical significance (all *p*>.060).

#### AMT scores and PANSS scores

In order to explore potential relationships between AMT performance and PANSS symptom ratings, correlations between AMT measures (again pooled across line condition as described above) and PANSS subscales were calculated for patients only. There was a significant negative correlation between reaction time on slow-long trials and positive symptom ratings (τ = −.367, *p* = .012) and a near-significant negative correlation between reaction time on slow-short trials and positive symptom ratings (τ = −.259, *p* = .053), indicating that higher positive symptom scores were associated with more negative differences between objective and estimated movement time, i.e. greater underestimation of movement time. All other correlations failed to reach significance (all *p* = .102).

## Discussion

The present study investigated the relationship between altered time perception on the AMT in schizophrenia and symptom manifestation in 22 patients and 22 controls. In accordance with the initial hypothesis, patients were impaired at estimating the time needed for a visual stimulus to complete a horizontal movement. More specifically, patients systematically underestimated the movement time on trials with high cognitive demand, i.e. on trials with slow movement speed and long distance. Crucially, impaired timing performance in patients was linked to symptom manifestation but not to cognitive impairment. PANSS positive symptom ratings were negatively correlated with AMT performance on the critical trial types, indicating that higher positive symptom scores were associated with greater underestimation of movement time on these trials.

At first glance, the observed underestimation of movement time is largely in line with previous findings suggesting an accelerated internal clock in schizophrenia that is presumed to be due to dopaminergic dysregulation [26], [28], [29]. This notion is supported by findings of impaired temporal processing in Parkinson’s disease, a disorder characterized by atrophy of the substantia nigra and resulting depletion of DA neurons. Patients with Parkinson’s disease have been reported to show a slowing of temporal processing that is at least partly eliminated by appropriate levodopa medication (for a review, see [45]).

More recently, the notion of a genuine time perception disorder in schizophrenia as reflected in increased speed of the internal clock has been challenged. Roy et al. [33] applied an innovative task requiring subjects to detect gradual changes in auditory tempo to estimate the natural frequency of the internal clock in patients with schizophrenia and healthy controls. While patients did exhibit increased variability on a control task involving reproduction of time intervals, there was no evidence for a faster natural frequency of the internal clock in patients compared to controls. Similarly, the present findings argue against a general timing deficit, performance being impaired only on cognitively demanding trials, i.e. on trials with long distances and slow movement speed. Contrary to the expectations, manipulation of cognitive load by means of the vanishing mark did not work as a corresponding increase in task difficulty when no mark was present did not differentially affect patients and controls.

The present pattern of results is in largely in line with the notion that temporal disturbances in schizophrenia may arise from disease-related cognitive dysfunction [33]. Indeed, temporal sensitivity for short (<1000 ms) or long (>1000 ms) intervals has been shown to correlate with measures of sustained attention or long-term memory, respectively, supporting the notion that time perception impairment in schizophrenia is influenced by neuropsychological dysfunction [32]. In the present sample, performance on the TMT suggested generally reduced processing speed and cognitive flexibility in patients. However, there was no clear link between impaired time estimation and executive dysfunction, suggesting that impaired temporal processing in schizophrenia cannot solely be ascribed to cognitive deficits, albeit doubtlessly being influenced by it. Rather, the pattern of results in the present study indicates that AMT performance was also modulated by symptom manifestation. PANSS positive symptom ratings were negatively correlated with AMT performance, i.e. higher scores on the positive symptom scale were associated with greater underestimation of movement time. Importantly, attentional and executive impairment has been reported to be particularly associated with negative symptoms [37]. In the present sample, PANSS negative symptom as well as general psychopathology ratings did not correlate significantly with AMT performance, further arguing against an interpretation of the present findings in terms of mere cognitive rather than timing disturbances in schizophrenia.

In line with the present findings of more pronounced impairment of temporal processing in patients with severe positive symptoms, a recent study has reported altered time interval reproduction in neurologically healthy individuals with high proneness to hallucinations [46]. High hallucination prone individuals estimated the duration of presentation of emotional – and particularly angry - faces as longer than low hallucination prone individuals. The authors suggest that the subjective experience of feeling exposed to threat for longer, which is in accordance with the notion of an accelerated internal clock, may be linked to the state of hypervigilance generally associated with positive symptoms of schizophrenia. Moreover, it has been proposed that impaired temporal processing may also contribute to other positive symptoms such as delusions and disorganized behavior [47]–[49]. Distortions in temporal structuring as reflected e.g. in perturbed asynchrony detection with enlarged time windows in which events are perceived as bound together [50] may also underlie an impaired sense of agency. Indeed, patients with schizophrenia fail to differentiate between the perception of self-produced and externally produced sensory signals, hence reporting self-produced tactile stimulation as tickly, whereas in healthy individuals, self-induced stimulation is perceptually attenuated [51], [52]. Furthermore, patients with schizophrenia show increased overall binding of actions and consequences [53], as do putatively prodromal individuals [54]. Accordingly, administration of ketamine, an established drug model for psychosis, was shown to increase action binding [55].

Interestingly, schizophrenia has recently also been associated with deficits in predictive timing [56]. Patients showed increased thresholds for the detection of phase shifts in rhythmic sound sequences. Notably, this deficit could not be attributed to changes in the internal clock, as time interval production was found to be preserved in patients. These findings are in line with the present results, since prediction constitutes a key component also on the AMT which requires subjects to anticipate when the stimulus reaches the target position. Systematic underestimation of movement time on the AMT thus corroborates impaired predictive timing in schizophrenia [57]. However, the present result pattern cannot unequivocally be ascribed to an accelerated internal clock, but merely adds to a growing body of evidence for a deficit in anticipatory and predictive mechanisms in schizophrenia [52], [56]–[58]. Interestingly, rather than suggesting that this deficit is general in nature, the present data indicate that it is critically modulated by cognitive demand.

It has to be noted that apart from the general cognitive status of the patients, medication may be a major factor influencing time processing in schizophrenia. A wide range of studies provide ample evidence for dopaminergic control of both speed of the internal clock and of the distribution of resources between time processing and other cognitive functions (for a review see [11]). Generally, dopamine agonists have been shown to increase clock speed, while antagonists decrease it [14]. Appropriate responses to antipsychotic medication in patients with schizophrenia have been reported to be associated with better time perception [59], [60]. Unfortunately, the impact of medication status was not systematically assessed in the present study. Future studies will have to investigate to what extent temporal processing in schizophrenia is modulated by antipsychotic medication.

Even though medication status and attention were not systematically assessed in the present study, the findings were obtained in a comparably large and carefully selected patient sample. The present study is among the first to investigate the impact of symptom manifestation on schizophrenia-related impairment patterns. Intriguingly, the findings of impaired predictive timing in schizophrenia may also yield important clinical implications: Prediction is a key factor underlying many activities and cognitive functions. As the AMT is a typical laboratory paradigm that cannot easily be compared to more complex real life settings, future studies should assess to what extent deficits in anticipatory and predictive mechanisms in schizophrenia may generalize to more ecologically valid settings, e.g. traffic or sports.

### Conclusion

Taken together, the present study provides further evidence for impaired temporal processing, and more specifically predictive timing, in schizophrenia. Patients underestimated the time a visual stimulus needed to complete a horizontal movement towards a target position. Crucially, in line with the role of dopamine both in schizophrenia and temporal processing, task performance was modulated by symptom manifestation. Impaired estimation of movement time was significantly correlated with PANSS positive symptom scores, higher scores being associated with stronger underestimation of movement time. Importantly, even though there was no clear link to disease-related cognitive dysfunction, patients were impaired only on cognitively more demanding trials. The present data thus support the notion of a deficit in anticipatory and predictive mechanisms in schizophrenia that is modulated both by symptom manifestation and by cognitive demand of the task at hand.

## References

[1] Miltner WHR (2008) Wenn Zeit zur Belastung wird. Psychologische und neurowissenschaftliche Aspekte. In: Kodalle KM, Rosa H, editors. Rasender Stillstand. Beschleunigung des Wirklichkeitswandels: Konsequenzen und Grenzen. Würzburg: Verlag Königshausen und Neumann. p 287–299.

[2] Troutwine R, O’Neal EC (1981) Volition, performance of a boring task and time estimation. Percept Mot Skills 52: 865–866. 7267259 10.2466/pms.1981.52.3.865

[3] Danckert JA, Allman AA (2005) Time flies when you’re having fun: temporal estimation and the experience of boredom. Brain Cogn 59: 236–245. 16168546 10.1016/j.bandc.2005.07.002

[4] Hagura N, Kanai R, Orgs G, Haggard P (2012) Ready steady slow: action preparation slows the subjective passage of time. Proc Biol Sci 279: 4399–4406. 22951740 10.1098/rspb.2012.1339 PMC3479796

[5] Treisman M (1963) Temporal discrimination and the indifference interval. Implications for a model of the “internal clock”. Psychol Monogr 77: 1–31. 10.1037/h0093864 5877542

[6] Gibbon J, Church RM, Meck WH (1984) Scalar timing in memory. Ann N Y Acad Sci 423: 52–77. 6588812 10.1111/j.1749-6632.1984.tb23417.x

[7] Meck WH (2006) Neuroanatomical localization of an internal clock: a functional link between mesolimbic, nigrostriatal, and mesocortical dopaminergic systems. Brain Res 1109: 93–107. 16890210 10.1016/j.brainres.2006.06.031

[8] Meck WH (2006) Frontal cortex lesions eliminate the clock speed effect of dopaminergic drugs on interval timing. Brain Res 1108: 157–167. 16844101 10.1016/j.brainres.2006.06.046

[9] Buhusi CV, Meck WH (2009) Relativity theory and time perception: single or multiple clocks? PLoS One 4: e6268. 19623247 10.1371/journal.pone.0006268 PMC2707607

[10] Buhusi CV, Meck WH (2009) Relative time sharing: new findings and an extension of the resource allocation model of temporal processing. Philos Trans R Soc Lond B Biol Sci 364: 1875–1885. 19487190 10.1098/rstb.2009.0022 PMC2685821

[11] Buhusi CV, Meck WH (2005) What makes us tick? Functional and neural mechanisms of interval timing. Nat Rev Neurosci 6: 755–765. 16163383 10.1038/nrn1764

[12] Meck WH (1983) Selective adjustment of the speed of internal clock and memory processes. J Exp Psychol Anim Behav Process 9: 171–201. 6842136

[13] Meck WH (1986) Affinity for the dopamine D2 receptor predicts neuroleptic potency in decreasing the speed of an internal clock. Pharmacol Biochem Behav 25: 1185–1189. 2880350 10.1016/0091-3057(86)90109-7

[14] Rammsayer TH (1993) On dopaminergic modulation of temporal information processing. Biol Psychol 36: 209–222. 8260566 10.1016/0301-0511(93)90018-4

[15] Lewis PA, Miall RC (2003) Brain activation patterns during measurement of sub- and supra-second intervals. Neuropsychologia 41: 1583–1592. 12887983 10.1016/s0028-3932(03)00118-0

[16] Macar F, Lejeune H, Bonnet M, Ferrara A, Pouthas V, et al (2002) Activation of the supplementary motor area and of attentional networks during temporal processing. Exp Brain Res 142: 475–485. 11845243 10.1007/s00221-001-0953-0

[17] Picton TW, Stuss DT, Shallice T, Alexander MP, Gillingham S (2006) Keeping time: effects of focal frontal lesions. Neuropsychologia 44: 1195–1209. 16271270 10.1016/j.neuropsychologia.2005.10.002

[18] Harrington DL, Haaland KY, Knight RT (1998) Cortical networks underlying mechanisms of time perception. J Neurosci 18: 1085–1095. 9437028 10.1523/JNEUROSCI.18-03-01085.1998 PMC6792777

[19] Coslett HB, Wiener M, Chatterjee A (2010) Dissociable neural systems for timing: evidence from subjects with basal ganglia lesions. PLoS One 5: e10324. 20428244 10.1371/journal.pone.0010324 PMC2859062

[20] Bonnot O, de Montalembert M, Kermarrec S, Botbol M, Walter M, et al (2011) Are impairments of time perception in schizophrenia a neglected phenomenon? J Physiol Paris 105: 164–169. 21803155 10.1016/j.jphysparis.2011.07.006

[21] Jaspers K (1973) Raum- und Zeiterleben. In Jaspers K, editor: Allgemeine Psychopathologie. Berlin: Springer. p 67–74.

[22] Arstila V (2011) Further steps in the science of temporal consciousness? In A. Vatakis et al., editors: Time and time perception 2010, LNAI 6789. Berlin: Springer. p 1–10.

[23] Kupke C. (2009) Begriff Zeit in der Psychopathologie. Berlin: Parodos.

[24] Vogeley K, Kupke C (2007) Disturbances of time consciousness from a phenomenological and a neuroscientific perspective. Schizophr Bull 33, 157–165. 10.1093/schbul/sbl056 PMC2632289 17105967

[25] Meck WH (1996) Neuropharmacology of timing and time perception. Brain Res Cogn Brain Res 3: 227–242. 8806025 10.1016/0926-6410(96)00009-2

[26] Tysk L (1983) Time estimation by healthy subjects and schizophrenic patients: a methodological study. Percept Mot Skills 56: 983–988. 6877984 10.2466/pms.1983.56.3.983

[27] Carroll CA, O’Donnell BF, Shekhar A, Hetrick WP (2009) Timing dysfunctions in schizophrenia as measured by a repetitive finger tapping task. Brain Cogn 71: 345–353. 19664870 10.1016/j.bandc.2009.06.009 PMC2783288

[28] Wahl OF, Sieg D (1980) Time estimation among schizophrenics. Percept Mot Skills 50: 535–541. 7375306 10.1177/003151258005000232

[29] Roy M, Grondin S, Roy MA (2012) Time perception disorders are related to working memory impairment in schizophrenia. Psychiatry Res 200: 159–166. 22862910 10.1016/j.psychres.2012.06.008

[30] Delevoye-Turrell Y, Wilquin H, Giersch A (2012) A ticking clock for the production of sequential actions: where does the problem lie in schizophrenia? Schizophr Res 135: 51–54. 22260961 10.1016/j.schres.2011.12.020

[31] Carroll CA, O’Donnell BF, Shekhar A, Hetrick WP (2009) Timing dysfunctions in schizophrenia span from millisecond to several-second durations. Brain Cogn 70: 181–190. 19282082 10.1016/j.bandc.2009.02.001

[32] Elvevag B, McCormack T, Gilbert A, Brown GD, Weinberger DR, et al (2003) Duration judgements in patients with schizophrenia. Psychol Med 33: 1249–1261. 14580079 10.1017/s0033291703008122

[33] Davalos DB, Kisley MA, Ross RG (2003) Effects of interval duration on temporal processing in schizophrenia. Brain Cogn 52: 295–301. 12907174 10.1016/s0278-2626(03)00157-x

[34] Lee KH, Bhaker RS, Mysore A, Parks RW, Birkett PB, et al (2009) Time perception and its neuropsychological correlates in patients with schizophrenia and in healthy volunteers. Psychiatry Res 166: 174–183. 19278734 10.1016/j.psychres.2008.03.004

[35] Davis KL, Kahn RS, Ko G, Davidson M (1991) Dopamine in schizophrenia: a review and reconceptualization. Am J Psychiatry 148: 1474–1486. 1681750 10.1176/ajp.148.11.1474

[36] Tanaka T, Tomotake M, Ueoka Y, Kaneda Y, Taniguchi K, et al (2012) Clinical correlates associated with cognitive dysfunction in people with schizophrenia. Psychiatry Clin Neurosci 66: 491–498. 23066766 10.1111/j.1440-1819.2012.02390.x

[37] Müller BW, Sartory G, Bender S (2004) Neuropsychological Deficits and Concomitant Clinical Symptoms in Schizophrenia. European Psychologist 9: 96–106.

[38] Reitan RM (1992) Trail Making Test. Manual for administration and scoring. Tucson, AZ: Reitan Neuropsychology Laboratory.

[39] Heaton HK, Cheloune GJ, Tally JL, Kay GG, Curtiss G (1993) Wisconsin Card Sorting Test manual revised and expanded. Odessa, FL: Psychological Assessment Resources.

[40] American Psychological Association (2000) Diagnostic and statistial manual of mental disorders (DSM-IV-TR). Washington, D.C.: Author.

[41] First M B, Spitzer R L, Gibbon M, Williams JBW (2002) Structured Clinical Interview for the DSM-IV-TR Axis I Disorders, Research Version, Patient Edition (SCID-I/P). New York: Biometrics Research, New York State Psychiatric Institute.

[42] Sheehan DV, Lecrubier Y, Sheehan KH, Amorim P, Janavs J, et al (1998) The Mini-International Neuropsychiatric Interview (M.I.N.I.): the development and validation of a structured diagnostic psychiatric interview for DSM-IV and ICD-10. J Clin Psychiatry 59 Suppl 2022–33. 9881538

[43] Kay SR, Fiszbein A, Opler LA (1987) The positive and negative syndrome scale (PANSS) for schizophrenia. Schizophr Bull 13: 261–276. 3616518 10.1093/schbul/13.2.261

[44] Lehrl S (2005) Mehrfachwahl-Wortschatz-Intelligenztest MWT-B. Balingen: Spitta Verlag.

[45] Allman MJ, Meck WH (2012) Pathophysiological distortions in time perception and timed performance. Brain 135: 656–677. 21921020 10.1093/brain/awr210 PMC3491636

[46] Coy AL, Hutton SB (2012) The influence of hallucination proneness and social threat on time perception. Cogn Neuropsychiatry 1–14 (epub ahead of print). DOI: 10.1080/13546805.2012.730994. 10.1080/13546805.2012.730994 23140175

[47] Ward RD, Kellendonk C, Kandel ER, Balsam PD (2012) Timing as a window on cognition in schizophrenia. Neuropharmacology 62: 1175–1181. 21530549 10.1016/j.neuropharm.2011.04.014 PMC3155658

[48] Carroll CA, Boggs J, O’Donnell BF, Shekhar A, Hetrick WP (2008) Temporal processing dysfunction in schizophrenia. Brain Cogn 67: 150–161. 18262701 10.1016/j.bandc.2007.12.005 PMC2512257

[49] Andreasen NC, Nopoulos P, O’Leary DS, Miller DD, Wassink T, et al (1999) Defining the phenotype of schizophrenia: cognitive dysmetria and its neural mechanisms. Biol Psychiatry 46: 908–920. 10509174 10.1016/s0006-3223(99)00152-3

[50] Giersch A, Lalanne L, Corves C, Seubert J, Shi Z, et al (2009) Extended visual simultaneity thresholds in patients with schizophrenia. Schizophr Bull 35: 816–825. 18359954 10.1093/schbul/sbn016 PMC2696372

[51] Blakemore SJ, Smith J, Steel R, Johnstone CE, Frith CD (2000) The perception of self-produced sensory stimuli in patients with auditory hallucinations and passivity experiences: evidence for a breakdown in self-monitoring. Psychol Med 30: 1131–1139. 12027049 10.1017/s0033291799002676

[52] Shergill SS, Samson G, Bays PM, Frith CD, Wolpert DM (2005) Evidence for sensory prediction deficits in schizophrenia. Am J Psychiatry 162: 2384–2386. 16330607 10.1176/appi.ajp.162.12.2384

[53] Haggard P, Martin F, Taylor-Clarke M, Jeannerod M, Franck N (2003) Awareness of action in schizophrenia. Neuroreport 14: 1081–1085. 12802207 10.1097/01.wnr.0000073684.00308.c0

[54] Hauser M, Moore JW, de Millas W, Gallinat J, Heinz A, et al (2011) Sense of agency is altered in patients with a putative psychotic prodrome. Schizophr Res 126: 20–27. 21112189 10.1016/j.schres.2010.10.031

[55] Moore JW, Cambridge VC, Morgan H, Giorlando F, Adapa R, et al.. (2012) Time, action and psychosis: Using subjective time to investigate the effects of ketamine on sense of agency. Neuropsychologia 1–8 (epub ahead of print). DOI: S0028–3932(12)00293-X. 10.1016/j.neuropsychologia.2012.07.005 PMC3562439 22813429

[56] Turgeon M, Giersch A, Delevoye-Turrell Y, Wing AM (2012) Impaired predictive timing with spared time interval production in individual with schizophrenia. Psychiatry Res 197: 13–18. 22497958 10.1016/j.psychres.2012.03.003

[57] Bays PM, Wolpert DM, Flanagan JR (2005) Perception of the consequences of self-action is temporally tuned and event driven. Curr Biol 15: 1125–1128. 15964278 10.1016/j.cub.2005.05.023

[58] Lalanne L, Van AM, Giersch A (2012) When predictive mechanisms go wrong: disordered visual synchrony thresholds in schizophrenia. Schizophr Bull 38: 506–513. 20876220 10.1093/schbul/sbq107 PMC3330002

[59] Angle HV (1973) Role of chlorpromazine in maintaining timing behavior in chronic schizophrenics. Psychopharmacologia 28: 185–194. 4694626 10.1007/BF00421403

[60] Ulferts J, Meyer-Lindenberg A, Gallhofer B (1999) Time discrimination: a comparison between healthy controls, unmedicated schizophrenics, zote-pine treated schizophrenics and schizophrenics treated with conventional neuroleptics. Neuropsychiatry 13: 133–138.

